# Treatment summaries for head and neck cancer survivors: a pilot study to improving patient recall and survivorship care plans

**DOI:** 10.1007/s00520-025-09406-9

**Published:** 2025-04-04

**Authors:** Shreyas G. Krishnapura, Aaron X. Lee, Tatsuki Koyama, James L. Netterville, Kyle Mannion, Alexander J. Langerman, Robert Sinard, Sarah L. Rohde, Michael C. Topf

**Affiliations:** 1 School of Medicine, Vanderbilt University, Nashville, TN USA; 2 Department of Biostatistics, Vanderbilt University Medical Center, Nashville, TN USA; 3 Department of Otolaryngology - Head and Neck Surgery, Vanderbilt University Medical Center, Nashville, TN USA

**Keywords:** Otolaryngology, Head and neck surgery, Cancer survival, Quality improvement, Patient safety, Survivorship care, Patient recall

## Abstract

**Background:**

The treatment summary (TS) remains underused in head and neck cancer (HNC). We determine if receiving a personalized TS following a definitive HNC treatment improves patient recall of treatment details.

**Method:**

A history questionnaire was administered to assess patient accuracy in reporting diagnosis and treatment-related details at baseline (pre-TS) and 1-month follow-up (post-TS). Statistical significance of accuracy changes was assessed using McNemar’s chi squared test.

**Results:**

One hundred participants were enrolled between January and December 2022. Ninety-one completed all surveys. Significant improvement in correct responses was noted for questions regarding cancer diagnosis type (94.5% post-TS, 78.0% pre-TS, *p* = 0.002), cancer diagnosis date (75.8% post-TS, 40.7% pre-TS, *p* < 0.001), T-stage (82.4% post-TS, 28.6% pre-TS, *p* < 0.001), and lymph node status (91.2% post-TS, 63.7% pre-TS, *p* < 0.001).

**Conclusion:**

TS may improve HNC survivors’ recall about their diagnosis and treatment in the short term. Patients view TS as beneficial to their survivorship care plans.

**Implications for Cancer Survivors:**

TS in survivorship care plans are understudied in HNC patients. This investigation will serve as a meaningful contribution to the literature, promote additional investigation, and encourage improvement of informative resources available to HNC survivors.

**Supplementary Information:**

The online version contains supplementary material available at 10.1007/s00520-025-09406-9.

## Introduction

Head and neck cancers (HNC) continue to pose a significant public health problem. In 2023, an estimated 54,540 people (39,290 men and 15,250 women) will be diagnosed with HNC [1]. As treatment improves, there has been a rise in HNC survivors [1, 2], encouraging a broadening of clinicians’ focus to include support for survivor care planning. Multiple societies, including the American Head and Neck Society [3], American College of Surgeons Commission on Cancer [4], and the American Cancer Society [5], have identified the need for comprehensive survivorship care planning. Within their recommendations, each society proposes that all cancer patients should receive a treatment summary (TS) form after therapy.

A TS includes detailed information about a patient’s tumor characteristics and cancer care; this information is crucial for patient knowledge and clinicians who provide follow-up care for cancer survivors. Unfortunately, incorporating TS in HNC survivorship care remains uncommon [6], and a recent survey revealed that only 43.1% of HNC programs currently distribute TS to their patients [7]. While studies have evaluated the effectiveness of survivorship care plans and TS in patients with breast and colorectal cancer [8–13], to our knowledge, no clinical studies have investigated the impact of TS on HNC patients’ recall of their treatment course.

All patients at our institution receive connections to support groups, information about therapy, lymphedema control pamphlets, counseling on the side effects of treatment, and other similar documents as part of their post-treatment care. Prior to this study, HNC patients at our institution did not receive a TS or similar document upon completion of therapy. In this pilot study, we implemented TS and distributed them to HNC patients who had completed definitive therapy. Patients were surveyed regarding details of their diagnosis and treatment regimen before and 1 month after receiving their TS. They were also asked their opinion on the usefulness of the cancer TS form at baseline and at a 1-month follow-up. It is our goal that by investigating TS in this population, we may work to promote an overall improvement in survivorship care planning.

## Methods

### Study design, cohort, and outcomes

This study was conducted at the Vanderbilt University Medical Center (VUMC) Department of Otolaryngology – Head and Neck Surgery. Eligible patients were identified from physician clinic schedules and tumor board lists and include those who are at least 1 month out from completing definitive therapy for their HNC, currently receive follow-up surveillance at in a head and neck cancer clinic at VUMC, and are able to read English. Patients with recurrent disease were included in the study. Patients were excluded if they did not meet eligibility criteria or if their treatment history could not be accurately determined from the electronic health record. The VUMC Institutional Review Board approved the study (IRB#: 211994). Informed consent was obtained from all individual participants included in the study. This study was performed in line with the principles of the Declaration of Helsinki.

### Treatment summary preparation and workflow

Using the electronic health record information, a personalized TS (See Supplemental Fig. 1) including names of healthcare providers, cancer diagnosis, pathology details, and treatment details was created for each participant by the study personnel the day before their clinic appointment. When necessary, the study personnel contacted other treatment facilities for treatment details for patients who received part of their cancer treatment elsewhere. TS only included a participants’ HNC cancer information and did not include details regarding non-HNC treatment. The TS was reviewed and approved by the treating surgical oncologist prior to distribution. All potential participants were approached regarding their interest in receiving a TS and participating in the study. All patients enrolled in the study were consented by the study personnel. No financial compensation was provided to participants.

**Fig. 1:**
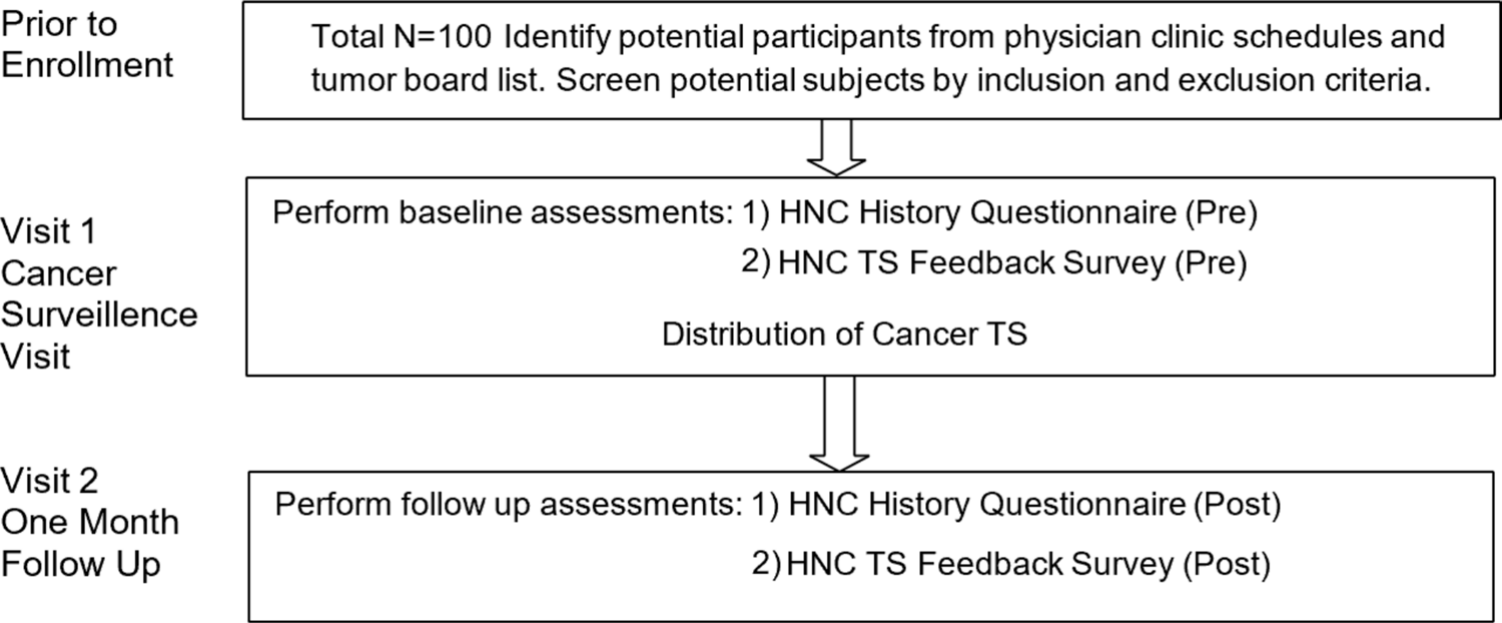
Study workflow (HNC—head and neck cancer; TS—treatment summary)

Following their scheduled clinic visit, patients were met by study personnel and introduced to the study. A 6-question HNC History Questionnaire (See Supplemental Fig. 2) was administered verbally with all questions and answer choices read aloud to assess patient accuracy in reporting diagnosis and treatment-related details at baseline (pre-TS). In the interest of respecting the patient’s time, the history questionnaire was kept short, and selectively tested details of their treatment history rather than asking for all details included in the TS. If a patient had prior history of more than one HNC, the patient was asked to report their recall of the most recent one. After completing the knowledge survey, a printed copy of their TS was provided, and study personnel walked each patient through the details included on the form and answered any questions from the patient and family. Afterward, a pre-TS patient feedback survey (see Supplemental Fig. 3) was administered verbally, asking patients about their expected usefulness of a TS. After 1 month, patients were contacted over the phone to administer a post-TS HNC history questionnaire (see Supplemental Fig. 4) and a post-TS feedback survey (see Supplemental Fig. 5) asking about their experience with the TS during the 1-month follow-up period (see Fig. 1 for the study workflow). Participants were allowed to use their TS during the 1-month follow-up interview.

### Data collection

Study data were collected and managed using REDCap electronic data capture tools hosted at VUMC [14, 15]. Variables of interest related to patient demographics and treatment history were manually extracted from the medical record including age, gender, race, ethnicity, alcohol history, smoking history, anatomic subsite of primary tumor, tumor T-stage, treatments received, and time from completion of treatment to receiving TS.

### Statistical analysis

Data for the study were extracted from the secure REDCap database and examined for uniformity and validity. Continuous variables are summarized with median and quartiles, and frequency and proportions are used to tabulate categorical variables. The accuracy of each treatment history question was determined for each patient by the study personnel. Analysis was limited to 91 patients who returned both pre- and post-TS surveys. Responses to treatment history questions were recorded as accurate or inaccurate; if the participant indicated “don’t know,” it was coded as inaccurate. Additionally, the date of diagnosis was categorized as correct if the respondent gave the precise month and year.

Total accurate reponses on both pre- and post-surveys were tallied for each of the six questions on the history questionnaire. An accuracy comparison on each question between pre-and post-TS was conducted using McNemar’s chi squared test with continuity correction. To investigate potential associations between the three outcomes (pre-treatment accuracy, post-treatment accuracy, and change in accuracy) and the following patient-level factors: age, gender, time from end of treatment to enrollment, and total number of treatments received, ordinal logistic regression models were fitted for each of the three outcomes. Given that the outcome encompasses only a limited number of possible values, an ordinal regression model, which does not require distributional assumptions about the residuals, was employed. Statistical significance was determined as *p* < 0.05. All analysis was performed using R4.2 [16].

## Results

Preparing each TS required dedicated time from the study personnel, in rare cases up to an hour per patient if certain treatment history was only available through requesting outside hospital records. Treatment summaries (TS) were provided to 100 patients between January and December 2022, of which 67.0% (*n* = 67) were male (Table 1). The median patient age was 66.0 years (55.0, 72.0). The majority (89.0%, *n* = 89) of participants were White. The participants had a history of HNC in a broad range of anatomic subsites, including oral cavity (32.0%, *n* = 32), oropharynx (23.0%, *n* = 23), and larynx/hypopharynx (21.0%, *n* = 21). Every T-stage was well-represented with 30.0% (*n* = 30) had a T1 tumor T-stage, 27.0% (*n* = 27) with T2, 20.0% (*n* = 20) with T3, and 21% (*n* = 21) with T4. The most common treatment modalities included surgery alone (31), surgery and adjuvant chemoradiation (20), and adjuvant radiation therapy (15). The median time between completing definitive treatment and receiving the TS was 16 months (quartiles: 9, 27).

**Table 1:** Patient demographics (SD, standard deviation; Q1, lower quartile; Q3, upper quartile)

	Overall (*N* = 100)
Patient gender	
Female	33 (33.0%)
Male	67 (67.0%)
Patient age (years)	
Mean (SD)	64.6 (11.9)
Median [Q1, Q3]	66.0 (55.0, 72.0)
Patient rrace	
Whte	89 (89.0%)
Black/African-American	8 (8.0%)
Other	3 (3.0%)
Ethnicity	
Hispanic or Latino	4 (4.0%)
Non-Hispanic	96 (96.0%)
Current or prior smoking history?	
No	29 (29.0%)
Yes	71 (71.0%)
Current or prior alcohol history?	
No	53 (53.0%)
Yes	47 (47.0%)
Anatomic subsite of primary tumor	
Alveolar ridge	4 (4.0%)
Hypopharynx	4 (4.0%)
Larynx	17 (17.0%)
Nasal cavity	6 (6.0%)
Oral cavity	35 (35.0%)
Oropharynx	23 (23.0%)
Paranasal sinus	1 (1.0%)
Salivary gland	4 (4.0%)
Skin	4 (4.0%)
Thyroid	2 (2.0%)
Tumor T-stage (AJCC 8th Edition)	
T1	30 (30.0%)
T2	27 (27.0%)
T3	20 (20.0%)
T4	21 (21.0%)
Unknown	2 (2.0%)
All treatments received	
Surgery only	31 (31.0%)
Chemotherapy only	1 (1.0%)
Radiation therapy only	2 (2.0%)
Immunotherapy only	0 (0.0%)
Surgery and chemotherapy	2 (2.0%)
Surgery and radiation therapy	15 (15.0%)
Chemotherapy and radiation therapy	20 (20.0%)
Surgery, chemotherapy, and radiation therapy	27 (27.0%)
Surgery, chemotherapy, radiation therapy, and immunotherapy	2 (2.0%)
Time from end of treatment to study enrollment (days)	
Mean (SD)	652 (594.47)
Median [Q1, Q3)	474 (272.50, 819.25)

**Fig. 2:**
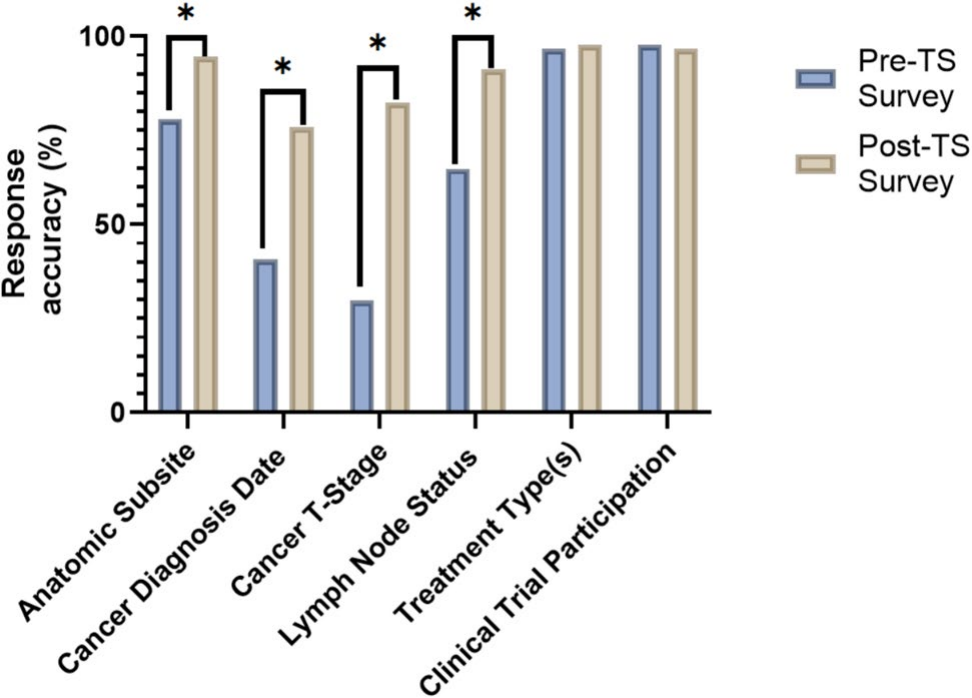
Cancer history questionnaire response accuracy proportions at both pre- and post-TS survey administration (TS, treatment summary, a single asterisk (*) indicates statistical significance)

Ninety-one patients completed both pre- and post-surveys. When asked to provide the anatomic location of their cancer diagnosis, accuracy was higher during the follow-up survey (78.0% vs. 94.5%) (Fig. 2). On the pre-survey, 40.7% (*n* = 37) correctly identified the date of their cancer diagnosis compared to 75.8% (*n* = 69) on the follow-up survey. For T-stage of cancer diagnosis, 29.7% (*n* = 27) answered correctly compared to 82.4% (*n* = 75) on the follow-up survey. For lymph node status, 64.8% (*n* = 59) correctly identified their diagnosis compared to 91.2% (*n* = 83) on the follow-up survey. There were minimal changes in response patterns between pre- and post-surveys for questions on treatment types and clinical trial participation.

Pre- to post-TS response accuracy difference by individual questions were calculated and presented in Fig. 2. Out of six questions, statistically significant improvement in patient accuracy was observed for the following four questions: cancer diagnosis type (*p* = 0.002), cancer diagnosis date (*p* < 0.001), cancer T-stage (*p* < 0.001), and lymph node status (*p* < 0.001). For patient treatment type(s) and clinical trial participation, very few patients had response accuracy changes from pre- to post-treatment survey administration, and no statistically significant improvement was observed.

For the pre-TS knowledge survey, older age significantly predicted total correct responses (odds ratio = 0.76, confidence interval = 0.63–0.93, *p* = 0.008 for 10-year increment). On the post-TS knowledge survey, there is insufficient evidence to suggest that there is an association between total post-TS response accuracy and patient age, patient gender, total treatments received, or time from end of treatment to enrollment in this study.

Patients were also given a survey to provide their thoughts on the utility of the TS (Table 2). A majority of patients showed agreement or strong agreement when asked if their TS led to better understanding of their cancer diagnosis and treatment on both pre- (93.4%, *n* = 85) and post-TS surveys (95.7%, *n* = 87). When asked if they would share their TS with their non-cancer doctor, a majority of patients agreed or strongly agreed on both pre- (78.1%, *n* = 71) and post-TS surveys (85.8%, *n* = 78). A majority of patients showed agreement or strong agreement when asked if their TS led to greater confidence when recalling cancer history to non-cancer doctors on both pre- (90.1%, *n* = 82) and post-TS surveys (86.8%, *n* = 79). When asked if a TS led to greater recall accuracy of cancer diagnosis and treatment, a majority of patients showed agreement or strong agreement on both pre- (96.7%, *n* = 88) and post-TS surveys (92.4%, *n* = 84). A majority of patients showed agreement or strong agreement when asked if their TS led to better communication of cancer diagnosis and treatment to loved ones on both pre- (95.6%, *n* = 87) and post-TS surveys (90.1%, *n* = 82). When asked if a TS would ease the transition from active treatment to survivorship, a majority of patients showed agreement or strong agreement on both pre- (75.9%, *n* = 69) and post-TS surveys (80.3%, *n* = 73). A majority of patients showed agreement or strong agreement when asked if their TS encouraged them to engage in their future health care on both pre- (92.3%, *n* = 84) and post-TS surveys (91.3%, *n* = 83). Additionally, patients were given the opportunity to provide free-text feedback on their overall experience with the treatment summary, which has been included in Supplemental Table 1.

**Table 2:** TS feedback survey response proportions at both pre- and post-TS administration

	Pre-TS survey	Post-TS survey
	Frequency (%)	Frequency (%)
Better understanding of cancer diagnosis and treatment		
Strongly agree	56 (61.5%)	42 (46.2%)
Agree	29 (31.9%)	45 (49.5%)
Neutral	5 (5.5%)	4 (4.4%)
Disagree	1 (1.1%)	0 (0%)
Strongly disagree	0 (0%)	0 (0%)
Information sharing with non-cancer doctors		
Strongly agree	38 (41.8%)	40 (44.0%)
Agree	33 (36.3%)	38 (41.8%)
Neutral	10 (11%)	9 (9.9%)
Disagree	10 (11%)	4 (4.4%)
Strongly disagree	0 (0%)	0 (0%)
Greater confidence when recalling cancer history to non-cancer doctors		
Strongly agree	60 (65.9%)	40 (44.0%)
Agree	22 (24.2%)	39 (42.9%)
Neutral	5 (5.5%)	8 (8.8%)
Disagree	4 (4.4%)	4 (4.4%)
Strongly disagree	0 (0%)	0 (0%)
Greater recall accuracy of cancer diagnosis and treatment		
Strongly agree	62 (68.1%)	43 (47.3%)
Agree	26 (28.6%)	41 (45.1%)
Neutral	1 (1.1%)	4 (4.4%)
Disagree	2 (2.2%)	3 (3.3%)
Strongly disagree	0 (0%)	0 (0%)
Better communication of cancer diagnosis and treatment to loved ones		
Strongly agree	54 (59.3%)	33 (36.3%)
Agree	33 (36.3%)	49 (53.8%)
Neutral	1 (1.1%)	5 (5.5%)
Disagree	2 (2.2%)	4 (4.4%)
Strongly disagree	1 (1.1%)	0(0%)
Ease of transition from active cancer treatment to survivorship		
Strongly agree	41 (45.1%)	32 (35.2%)
Agree	28 (30.8%)	41 (45.1%)
Neutral	15 (16.5%)	12 (12.2%)
Disagree	6 (6.6%)	6 (6.6%)
Strongly disagree	1 (1.1%)	0 (0%)
Encouragement towards proactivity in engaging in personal health care		
Strongly agree	53 (58.2%)	42 (46.2%)
Agree	31 (34.1%)	41 (45.1%)
Neutral	3 (3.3%)	4 (4.4%)
Disagree	4 (4.4%)	4 (4.4%)
Strongly disagree	0 (0%)	0 (0%)

## Discussion

Our study demonstrates that treatment summaries (TS) may improve cancer survivors’ recall of their diagnosis and treatment in the short term. Significant improvement in accuracy was shown in the recall of cancer diagnosis, date of diagnosis, staging, and lymph node involvement. Age, gender, number of treatments received, and time since completion of therapy did not impact post-TS accuracy, while older patients performed better on the pre-survey.

A complete survivorship care plan includes a summary of the patient’s cancer treatment history as well as any follow-up appointments, effects from treatments, and other relevant resources. For this pilot study, we chose to focus on the TS component of the care plan. The efficacy of TS has been studied extensively in other cancer populations. In a study with breast and colorectal cancer patients, patients reported a significant improvement in accuracy for recalling the stage of disease and treatment regimen after receiving a TS [12]. However, their study involved mailing the form and surveys to participants, whereas our study was designed to review the TS in person with each patient. This may play a role in the high accuracy changes we noted and supports the creation of a dedicated survivorship appointment following the completion of definitive treatment to further improve patient understanding of their cancer. In another study on survivorship care plans and TS in colorectal cancer patients, all patients reported satisfaction with having a comprehensive summary of their treatment and a “roadmap” for what they could expect regarding follow-up care. In addition, all survivors noted that TSs enhanced their understanding of their cancer and treatment history [13], supporting our findings.

Our results highlight the value of TS in improving patients’ recall of their cancer history. Our pre-TS survey results showed that most patients were unaware of the date of their diagnosis or staging information. Furthermore, 22% could not accurately recall the anatomic subsite of their cancer, and 36% were uncertain of their lymph node status. These results may highlight an unmet need for more thorough patient education regarding their diagnosis and history. Prior studies in the broader oncologic population have shown an increasing interest among survivors for additional information regarding their cancer [17]. Furthermore, meeting the informational needs of survivors has been shown to improve patient self-efficacy and psychological well-being [18, 19]. To our knowledge, there has been limited investigation of patient knowledge/recall of their cancer in the HNC population [20]. This investigation found that our patient population recalls their cancer history less accurately than those in previous studies, highlighting the need for TS and improved education during postoperative surveillance.

Many participants commented on the usefulness of having all of their information on one page, noting the same information was provided at prior visits but in multiple locations within their health records. Some said they had tried to keep track of their information but became overwhelmed by the volume and complexity (see Supplemental Table 1 containing free-response patient feedback). This feeling of informational excess has been well-reported among cancer patients [21–23], including the HNC population [24, 25], and supports the need for a concise and accessible summary. Additionally, patients noted that the TS included information about their cancer that they would not have known to ask and prompted additional questions.

Not all patients found the TS beneficial. Some patients stated that the TS provided no new information, and others were uncertain of the form’s utility. Additionally, though a majority of patients expressed a general agreement (either “strongly agree” or “agree”) of the TS’s utility before and after receiving the form, we were surprised to see a decrease in agreement level (from “strongly agree” to “agree”) for nearly all post-TS questions. A prior study investigating survivorship care plans (SCP) in HNC patients showed that most respondents were unsure of the utility of SCP and unable to locate their SCP 3 years after they were provided [25]. Patients in this study also noted the challenges in keeping track of the TS, with many stating that they had misplaced the document by the time of the post-TS survey. This brings up the need to consider more accessible modes of presenting the TS, such as an electronic version or one uploaded to their EMR.

The benefit of the TS for cancer patients has been called into question, citing concerns for significant time investment as well as the potential discrepancy between the value to physicians versus the value to patients [13, 26]. The TS is both a patient education tool as well as a provider-provider communication tool and as such, information that is pertinent to one group may not be as memorable to the other. This may explain the decrease in agreement-level for patients on the post-TS survey, as patients may have appreciated a different set of information to what was presented. For example, one patient commented that they would like to see information from their physical therapy sessions on the TS. Finally, it was not assessed whether the information included in the TS was presented in a way that is accessible to all patients, including those with lower health literacy. In future implementations, it would be prudent to codesign the TS with a wide group of patients and primary care providers, thereby improving the accessibility and relevance of the material. Feedback from patients and even consultation of validated quality-of-life metrics such as the Patient Concerns Inventory (PCI) will allow future versions of the TS to better address the complex informational needs of our patients [27].

Despite strong advocacy from the ACS and related initiatives, a recent American Head and Neck Society (AHNS) members survey revealed that less than half currently provide TS to HNC survivors [7, 28]. It is our goal that this pilot study serves as the foundation for designing better processes for the promotion of survivorship in cancer patients at this institution. However, determining the optimal workflow has been challenging in both academic and private settings, further complicating the uniform adoption of TS [7]; therefore, a designated role for preparing TS must be established in the treatment pathway to promote the initiative’s sustainability. One possibility is to include a role for preparing TS during Tumor Board meetings as much of the key information will be discussed during these sessions. Furthermore, if physicians, allied health professionals, and patients do not uniformly support the survivorship care planning initiative, then the TS’s value will remain uncertain. Given the frequent fragmentation of care provision in contemporary oncology, a system that reliably summarizes and communicates critical aspects of diagnosis and prior treatment may be invaluable, even from the vantage of patient safety [29]. The document may inform both survivors and their providers of key treatment history details and about any medical concerns that could impact their future health. Cancer survivors and primary care providers endorse the idea of TS and believe their wide availability would be helpful [30–33].

To our knowledge, this is the first study on the efficacy of TS in improving the recall of HNC patients. This study’s strengths are its relatively large sample size and its high patient response rate. However, our results are from one tertiary care center and may not apply to different healthcare settings. This study enrolled on average close to 2 years after completion of treatment. Though it is valuable to see data from patients at any stage of survivorship, future studies with a stronger focus on patients transitioning from active treatment to survivorship (e.g., using a set or constant time of enrollment) will be important. Furthermore, this study was limited in that no formal control group was used, instead allowing patients to serve as their control. Hence, the possibility that factors other than receiving a TS (such as other parts of their care plan) influenced a patient’s recall of their cancer history or transition to survivorship cannot be entirely ruled out. This pilot is an uncontrolled study and though it cannot demonstrate efficacy, it may provide data to serve as a useful foundation for future, more rigorous research. Additionally, the follow-up period was limited to 1 month, and therefore our study does not measure the influence of TS on long-term knowledge improvement. However, it could be argued that since patients may keep the TS and use it for reference, the TS may aid in their recall for months or years to come. Despite these limitations, we believe this study demonstrates feasibility of implementing TS in an otolaryngology clinic, adds to the current literature, and sets the stage for further investigation into patient education and self-efficacy in the HNC population.

## Conclusion

Treatment summaries (TS) may improve patients’ cancer and treatment history recall in the short term. Future studies will include a randomized trial with a formal control group and a more extended follow-up period that would focus on broader outcome measures, including adherence to follow-up and surveillance guidelines (tracking no-show rates), and patient satisfaction using surveys such as the Consumer Assessment of Healthcare Providers and Systems (CAHPS) [34]. Future efforts will also explore the formalization of a role in the clinic or Tumor Board workflow to create TS, encouraging sustainability and support for this valuable endeavor.

## Implications for cancer survivors

The use of treatment summaries as a component of survivorship care plans is beneficial in other cancer populations though is understudied in HNC survivors. This population faces unique challenges following treatment of their cancer, though the low number of current HNC providers who regularly provide TS to their patients is indicative of a critical gap in comprehensive survivorship care planning. It is our goal that this investigation will serve as a meaningful contribution to the literature, encourage additional investigation, and support that all HNC survivors should have a TS as part of their survivorship care plan.

## Supplementary Information

Below is the link to the electronic supplementary material.

Supplementary file1 (DOCX 165 KB)

Supplementary file2 (PDF 35 KB)

Supplementary file3 (PDF 36 KB)

Supplementary file4 (PDF 89 KB)

Supplementary file5 (PDF 53 KB)

Supplementary file6 (DOCX 17 KB)

## Data Availability

No datasets were generated or analysed during the current study.
