# Supplementary figures and images for: Treatment summaries for head and neck cancer survivors: a pilot study to improving patient recall and survivorship care plans
Source: Support Care Cancer. 2025 Apr 4;33(4):351. doi: 10.1007/s00520-025-09406-9 (PMC11971132; doi:10.1007/s00520-025-09406-9)

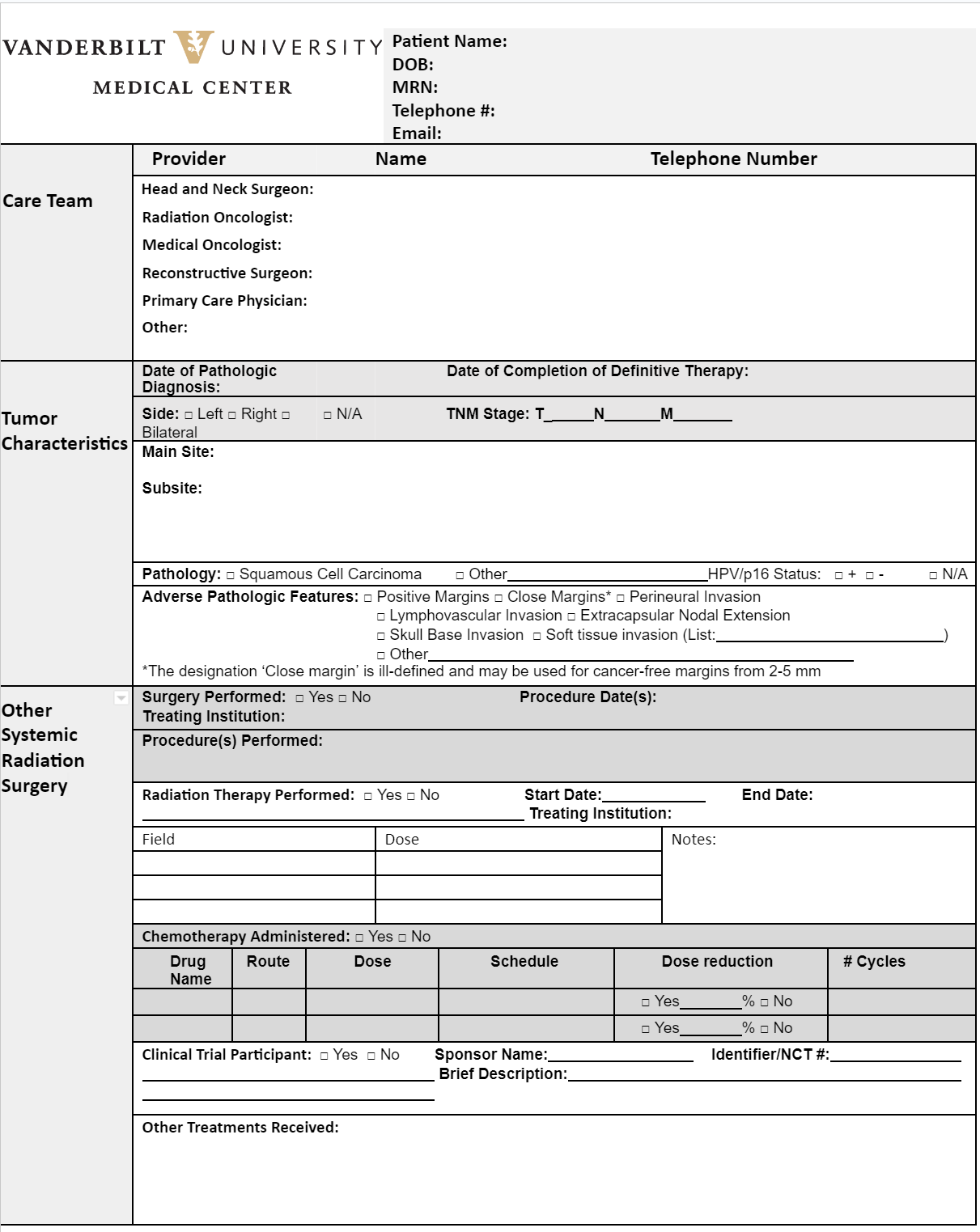


**Supplemental figure 1:** Treatment Summary (TS) example template

Supplement: Supplementary file 1 — Supplementary file1 (DOCX 165 KB) [file 520_2025_9406_MOESM1_ESM.docx]
