# Supplementary material for: Treatment summaries for head and neck cancer survivors: a pilot study to improving patient recall and survivorship care plans
Source: Support Care Cancer. 2025 Apr 4;33(4):351. doi: 10.1007/s00520-025-09406-9 (PMC11971132; doi:10.1007/s00520-025-09406-9)
Supplement: Supplementary file 2 — Supplementary file2 (PDF 35 KB) [file 520_2025_9406_MOESM2_ESM.pdf]

Head and Neck Cancer History Questionnaire - Baseline

Please complete the survey below.

Thank you!

What type of Head and Neck Cancer have you been diagnosed with?

- ☐ Skin (Scalp/Face/Neck)
- ☐ Larynx (Supraglottic Larynx/Glottic Larynx/Suglottic Larynx)
- ☐ Hypopharynx (Piriform Sinus/Posterior Pharyngeal Wall)
- ☐ Alveolar Ridge (teeth/jaw)
- ☐ Oral Cavity (Lip/Floor of Mouth/Buccal Mucosa/Retromolar Trigone/Hard Palate/Tongue)
- ☐ Oropharynx (Tonsils/Base of Tongue/Soft Palate)
- ☐ Salivary Gland (Parotid/ Submandibular)
- ☐ Nasal Cavity/Paranasal Sinus/Nasopharynx
- ☐ Thyroid
- ☐ Parathyroid
- ☐ I don't know

What month and year were you diagnosed with cancer (MM/YYYY)?

____

What T-stage of Head and Neck Cancer did you have?

- ☐ Stage 0 (carcinoma in situ)
- ☐ Stage 1
- ☐ Stage 2
- ☐ Stage 3
- ☐ Stage 4
- ☐ I don't know
- ☐ Unknown

Was cancer found in your lymph nodes?

- ☐ Yes
- ☐ No
- ☐ I don't know
- ☐ They did not test for this

Which of the following treatments have you received (check all that apply)?

- ☐ Surgery
- ☐ Chemotherapy
- ☐ Immunotherapy
- ☐ Radiation Therapy
- ☐ I don't know

Did you participate in any clinical trials for the treatment of your cancer?

- ◯ Yes
- ◯ No
- ◯ I don't know

What sources of information did you use to complete these questions?

- ☐ My own knowledge and memory
- ☐ I asked my spouse or children
- ☐ I checked records or notes that I have of my medical history
- ☐ Other

Please specify what other sources of information did you use to complete these questions?

____
