# Supplementary material for: Treatment summaries for head and neck cancer survivors: a pilot study to improving patient recall and survivorship care plans
Source: Support Care Cancer. 2025 Apr 4;33(4):351. doi: 10.1007/s00520-025-09406-9 (PMC11971132; doi:10.1007/s00520-025-09406-9)
Supplement: Supplementary file 3 — Supplementary file3 (PDF 36 KB) [file 520_2025_9406_MOESM3_ESM.pdf]

Head and Neck Cancer Treatment Summary - Initial Survey

Please complete the survey below.

Thank you!

1) A treatment summary would help me better understand my cancer diagnosis and treatment
- ◯ Strongly agree
- ◯ Agree
- ◯ Neutral
- ◯ Disagree
- ◯ Strongly Disagree

2) I would take my treatment summary when going to see my non-cancer doctors
- ◯ Strongly agree
- ◯ Agree
- ◯ Neutral
- ◯ Disagree
- ◯ Strongly Disagree

3) A treatment summary would help me feel more confident when recalling my cancer history to my non-cancer doctors or to any future doctors.
- ◯ Strongly Agree
- ◯ Agree
- ◯ Neutral
- ◯ Disagree
- ◯ Strongly Disagree

4) A treatment summary will help me accurately remember details about my cancer diagnosis and treatment
- ◯ Strongly Agree
- ◯ Agree
- ◯ Neutral
- ◯ Disagree
- ◯ Strongly Disagree

5) A treatment summary would help me better communicate details about my cancer diagnosis and treatment to my family and friends
- ◯ Strongly Agree
- ◯ Agree
- ◯ Neutral
- ◯ Disagree
- ◯ Strongly Disagree

6) A treatment summary would help ease the transition from active cancer treatment to survivorship
- ◯ Strongly Agree
- ◯ Agree
- ◯ Neutral
- ◯ Disagree
- ◯ Strongly Disagree

7) Having a treatment summary would encourage me to ask more questions and engage in my future health care
- ◯ Strongly Agree
- ◯ Agree
- ◯ Neutral
- ◯ Disagree
- ◯ Strongly Disagree

8) A list of resources that the hospital offers to support my medical/social health would be helpful to me
- ◯ Strongly Agree
- ◯ Agree
- ◯ Neutral
- ◯ Disagree
- ◯ Strongly Disagree
