# Supplementary material for: Treatment summaries for head and neck cancer survivors: a pilot study to improving patient recall and survivorship care plans
Source: Support Care Cancer. 2025 Apr 4;33(4):351. doi: 10.1007/s00520-025-09406-9 (PMC11971132; doi:10.1007/s00520-025-09406-9)
Supplement: Supplementary file 4 — Supplementary file4 (PDF 89 KB) [file 520_2025_9406_MOESM4_ESM.pdf]

Head and Neck Cancer History Questionnaire - POST

Please complete the survey below.

Thank you!

What type of Head and Neck Cancer have you been diagnosed with?	☐ Skin (Scalp/Face/Neck)<br>☐ Larynx (Supraglottic Larynx/Glottic Larynx/Suglottic Larynx)<br>☐ Hypopharynx (Piriform Sinus/Posterior Pharyngeal Wall)<br>☐ Alveolar Ridge (teeth/jaw)<br>☐ Oral Cavity (Lip/Floor of Mouth/Buccal Mucosa/Retromolar Trigone/Hard Palate/Tongue)<br>☐ Oropharynx (Tonsils/Base of Tongue/Soft Palate)<br>☐ Salivary Gland (Parotid/ Submandibular)<br>☐ Nasal Cavity/Paranasal Sinus/Nasopharynx<br>☐ Thyroid<br>☐ Parathyroid<br>☐ I don't know
What month and year were you diagnosed with cancer (MM/YYYY)?	________________________
What T-stage of Head and Neck Cancer did you have?	☐ Stage 0 (carcinoma in situ)<br>☐ Stage 1<br>☐ Stage 2<br>☐ Stage 3<br>☐ Stage 4<br>☐ I don't know<br>☐ Unknown
Was cancer found in your lymph nodes?	☐ Yes<br>☐ No<br>☐ I don't know<br>☐ They did not test for this
Which of the following treatments have you received (check all that apply)?	☐ Surgery<br>☐ Chemotherapy<br>☐ Immunotherapy<br>☐ Radiation Therapy<br>☐ I don't know
Did you participate in any clinical trials for the treatment of your cancer?	○ Yes<br>○ No<br>○ I don't know
What sources of information did you use to complete these questions?	☐ My own knowledge and memory<br>☐ I asked my spouse or children<br>☐ I checked records or notes that I have of my medical history<br>☐ Other
Please specify what other sources of information did you use to complete these questions?	________________________
