# Supplementary material for: Treatment summaries for head and neck cancer survivors: a pilot study to improving patient recall and survivorship care plans
Source: Support Care Cancer. 2025 Apr 4;33(4):351. doi: 10.1007/s00520-025-09406-9 (PMC11971132; doi:10.1007/s00520-025-09406-9)
Supplement: Supplementary file 5 — Supplementary file5 (PDF 53 KB) [file 520_2025_9406_MOESM5_ESM.pdf]

Head and Neck Cancer Treatment Summary - POST

Please complete the survey below.

Thank you!

1) My treatment summary helped me better understand my cancer diagnosis and treatment
   - ◯ Strongly agree
   - ◯ Agree
   - ◯ Neutral
   - ◯ Disagree
   - ◯ Strongly Disagree

2) I have shared and will continue to share my treatment summary with my non-cancer doctors
   - ◯ Strongly agree
   - ◯ Agree
   - ◯ Neutral
   - ◯ Disagree
   - ◯ Strongly Disagree

3) My treatment summary helped me feel more confident when recalling my cancer history to my non-cancer doctors or to any future doctors
   - ◯ Strongly Agree
   - ◯ Agree
   - ◯ Neutral
   - ◯ Disagree
   - ◯ Strongly Disagree

4) My treatment summary helped me accurately remember details about my cancer diagnosis and treatment
   - ◯ Strongly Agree
   - ◯ Agree
   - ◯ Neutral
   - ◯ Disagree
   - ◯ Strongly Disagree

5) My treatment summary helped me better communicate details about my cancer diagnosis and treatment to my family and friends
   - ◯ Strongly Agree
   - ◯ Agree
   - ◯ Neutral
   - ◯ Disagree
   - ◯ Strongly Disagree

6) My treatment summary helped ease the transition from active cancer treatment to survivorship
   - ◯ Strongly Agree
   - ◯ Agree
   - ◯ Neutral
   - ◯ Disagree
   - ◯ Strongly Disagree

7) Having my treatment summary has encouraged me to ask more questions and engage in my future health care
   - ◯ Strongly Agree
   - ◯ Agree
   - ◯ Neutral
   - ◯ Disagree
   - ◯ Strongly Disagree

8) A list of resources that the hospital offers to support my medical/social health has been helpful to me
   - ◯ Strongly Agree
   - ◯ Agree
   - ◯ Neutral
   - ◯ Disagree
   - ◯ Strongly Disagree

9) Along with the details already found in your treatment summary, what additional information would you find helpful?

   ____

10) Along with the details already found in the resource list, what additional information would you find helpful?

____
