# Supplementary material for: Treatment summaries for head and neck cancer survivors: a pilot study to improving patient recall and survivorship care plans
Source: Support Care Cancer. 2025 Apr 4;33(4):351. doi: 10.1007/s00520-025-09406-9 (PMC11971132; doi:10.1007/s00520-025-09406-9)
Supplement: Supplementary file 6 — Supplementary file6 (DOCX 17 KB) [file 520_2025_9406_MOESM6_ESM.docx]

| **Supplemental Table 1:** Patient free-response comments after receiving the treatment summary (TS) |
| --- |
| Very informative, very happy with the treatment summary and have already used it with my non-cancer. No formative comments. |
| I enjoyed having all of the information in one place because it can be overwhelming at times. I would have appreciated information at the bottom regarding what concerning features to lookout for |
| The treatment summary was thorough and I was able to help others in my life become more aware of my type of cancer |
| I have not used the treatment summary since the last visit. I don’t feel that I have difficulty in remembering my cancer history and diagnosis. Maybe if I had more extensive surgery or chemo/radiation history then I would have found more use from it |
| A lot of stuff that I couldn't remember or things that I wouldn't know to ask that was included on the treatment summary. Easy to read. Would appreciate even more information if possible and information in more detail |
| I have had many other surgeries beyond just my head and neck cancer. For just my head and neck cancer, it was very adequate. For example, I did not know my cancer stage and now I do. I appreciate that I have another useful form that I can present to my other doctors. |
| I appreciated the level of detail included and would not have asked for more information. It has helped me understand my diagnosis better. |
| Well constructed, good amount of information without too much excess. Would have appreciated being given earlier (right after her surgery if possible) |
| Loved every part of it. Only part I would have liked is that I couldn't access the treatment summary in my MyHealth app or other online version. Would appreciate this and to have the document in such a way that it could be shared with my out-of-Vanderbilt providers. |
| I received a therapy consult post-operatively and would have appreciated the suggestions of the therapist to be included in the treatment summary so that I could keep up those suggestions |
| Found it to be very comprehensive for my cancer diagnosis and treatment journey |
| I would appreciate a digital version of the treatment summary, making it easier to access. It is well-made and would have helped me right after I completed treatment; there were points where I was not able to remember all of my treatments and this TS would have helped for that |
| I misplaced the treatment summary and so would have appreciated it if it could also have been emailed or made into a digital copy |
| Used the treatment summary when answering questions today. Found it helpful. Easy to read. Nice to be made aware of things I wasn't aware of. Sometimes, I dont know what things mean, like what does staging mean? I know I can read it off the sheet, but what is that? It does make me ask more questions, but would like to have a key/legend about what complicated terms mean |
| It was very helpful to have all of my information in one place because it can get confusing after so many years |
| Took it when I saw my non-cancer doctor and it was very helpful to answering the doctor's intake questions. Awesome. |
| Found the information comprehensive and appreciated seeing all of my treatments on one page. Made it easier to read. Very helpful |
| Make it applicable for applying for disability insurance |
| I'm not very good with medical stuff and it is helpful to have everything on this one page because I get very overwhelmed when people ask me questions about specific chemo drugs |
| Laid out very well. Helps me to get over everything that's happened a little better because there is a finality in getting the summary to mark the end of treatment |
| Would appreciate having it sooner after treatment. Would have been able to understand my treatments a lot better. Still a lot of benefit even getting the summary so many years later |
| Dont have strong feelings toward the treatment summary. Personally dont find it to be helpful, however, recognize that others may get more use out of it |
| I finds computer-based things more beneficial, but can imagine older patients and someone who has had more treatments to be more beneficial. I like having my treatment history all on one page, and would like to scan the treatment summary into their chart as well for easy access |
| My daughter in law works for ACS and she really likes this. It can be really overwhelming when you first get treated for cancer and so this is very helpful to break things down in an easy-to-read one-pager. Top-notch. My family really appreciated it too |
| Reason I didn't find it helpful is that I have been keeping my doctors up to date all along and so it didn't add a lot of additional information. It was nice to have all of my doctors and their phone numbers at the top of the page and it was helpful to have the staging information as that was never discussed with me. |
| Include more information about the cancer like natural history and side effects |
| Personally, want to just "get through" treatment rather than get into technical details. I do appreciate that having the treatment summary means that I don’t need to spend time or effort learning the information itself, but I’m just not as interested in this information |
| Very awesome as it is so easy to forget everything that has happened with all of the treatments I have had. Showed the treatment summary to family and it is helpful as a reference point for me to verify that my memory is correct. Super! |
| Met a lot of people during this process and it can be difficult to keep track of everything that was going on. This treatment summary is a good step in the right direction to help in remembering little details |
| Really enjoyed the treatment summary. The most helpful part of this is the staging information. I had not seen my staging information anywhere else and was very confused about that until I received this treatment summary. |
| Very helpful in knowing the stage of cancer, especially for someone who is a little older and has trouble remembering |
| Enjoyed the treatment summary a lot |
| I liked having everything on one page and it helped me to understand my cancer better. |
| This has been extremely helpful. It has been very difficult to keep things concise, especially for someone who has had so many recurrences of cancer and different treatment modalities for each |
| This helped to fill in knowledge gaps that I didn't know I had |
| A visual depiction of the tumor would have been nice to see. A representation of the size of the tumor. Really good document and very valuable. The people in my life are non-medical and really want to hear about my treatment history. I can summarize my history a lot better with the treatment summary. Very difficult to do if you just have the information in a bunch of different places. This is done in such a way that it is a helpful visual communication of my history. Therapeutic discussions with other cancer survivors can be made easier with this. |
| Like having all the information on one page |
| Didn't find it helpful to me personally. Did not see the need to know these details of my history as I’ve just put my cancer behind me |
| Show it to my family and it is a lot more helpful than I ever thought it could be for just a single piece of paper. I do wish that I had gotten it earlier but I it is remarkable. |
| Found this to be excellent. I had tried to collect all of the information about the cancer but everything was so piecemail that it was impossible to keep up |
| Learned many new things about my cancer that I never knew about before like I didnt know it was hypopharynx cancer instead of larynx |
| Was pretty knowledgeable about my cancer history but still appreciated having a "cheat sheet" to have on hand |
